# Mechanistic insights into the role of PEDF-R (PNPLA2) in photoreceptors

**DOI:** 10.1042/BSR20250121

**Published:** 2026-07-09

**Authors:** S. Patricia Becerra

**Affiliations:** NIH-NEI, Laboratory of Retinal Cell and Molecular Biology, Section of Protein Structure and Function, 6 Center Drive, Bethesda, MD 20892, U.S.A.

**Keywords:** lipids, neuroprotection, PEDF-R, phospholipases, photoreceptors, PNPLA2

## Abstract

Photoreceptors are highly specialized neurons that depend on continuous membrane renewal and tightly regulated lipid homeostasis to maintain their visual function. Age-associated disruptions of these processes increase cellular stress and contribute to photoreceptor degeneration. Pigment epithelium-derived factor (PEDF) is a potent neuroprotective factor in the retina, and the identification of its receptor, PEDF-R (PNPLA2), has provided key mechanistic insight into how PEDF signaling is coupled to lipid metabolism. The present review examines the molecular and cellular mechanisms underlying PEDF-R function in photoreceptors and the retinal pigment epithelium (RPE). PEDF-R acts as a multifunctional enzyme with phospholipase and lipase activities that link extracellular PEDF binding to intracellular lipid remodeling. Through these activities, PEDF-R has been associated with processes essential for photoreceptor survival such as membrane phospholipid turnover, mitochondrial integrity, calcium homeostasis, and redox balance. In addition, PEDF-R contributes to retinoid metabolism and lipid processing associated with outer-segment renewal in the RPE. We further discuss how disruption of the PEDF-PEDF-R pathway impairs lipid homeostasis, promotes oxidative and inflammatory stress, and increases susceptibility to age-related retinal degeneration. These insights position PEDF-R as a key contributor to photoreceptor homeostasis and a potential therapeutic target for preserving retinal function in aging and disease.

## Introduction

Photoreceptors are among the most metabolically demanding and structurally specialized cells in the nervous system, requiring continuous membrane renewal and tightly controlled lipid homeostasis to sustain visual function [1]. Pigment epithelium-derived factor (PEDF) has long been recognized as a potent neuroprotective factor in the retina exerting neurotrophic and anti-angiogenic effects in preclinical models of retinal degeneration and vascular disorders [2]. PEDF is capable of promoting photoreceptor survival in preclinical models, and PEDF-based gene therapy approaches are being advanced into early clinical trials for retinitis pigmentosa [3], highlighting its translational potential. However, the molecular mechanisms underlying its protective actions have only gradually emerged. Patatin-like phospholipase A (PNPLA) proteins are lipid hydrolases involved in lipid metabolism and membrane homeostasis, share the conserved patatin domain originally identified in potato patatin, and exhibit distinct substrate specificities that include triacylglycerols, phospholipids, and retinyl esters (REs). The identification of PEDF receptor (PEDF-R), encoded by *PNPLA2* and also known as adipose triglyceride lipase (ATGL) or desnutrin, revealed an unexpected link between PEDF signaling and lipid metabolism [4]. Age-associated alterations in lipid handling and membrane maintenance place additional stress on photoreceptors, suggesting that the PEDF–PEDF-R pathway contributes to sustaining photoreceptor homeostasis across the lifespan. The present review provides mechanistic insights into the PEDF-R function in photoreceptors, with emphasis on lipid metabolism, neuroprotection, and aging.

## Identification of PEDF-R (PNPLA2) in the eye and retina

PEDF-R was initially characterized as a patatin-like phospholipase with triglyceride lipase activity, central to systemic lipid metabolism [5–7]. Its subsequent identification as a PEDF-binding protein expanded its functional scope beyond adipose tissue [4]. Early studies showing PEDF-R expression in ocular tissues, including the retina, suggested potential neuronal roles. This section traces the discovery of PEDF-R in the eye and highlights its conceptual shift from a lipid-metabolizing enzyme to a mediator of PEDF-dependent signaling in the retina.

In the early 1990s, PEDF was identified in conditioned media from retinal pigment epithelium (RPE) cell cultures as a secreted factor capable of promoting neuronal differentiation in retinoblastoma cells [8]. Subsequent work demonstrated that PEDF promotes survival of retinal neurons and other central and peripheral nervous system cells [9–11]. The gene encoding PEDF was expressed in young cells but not expressed in senescent cells, linking PEDF expression to cellular health and aging [12]. Sequence analysis revealed that PEDF belongs to the serine protease inhibitor (serpin) superfamily, a group of proteins sharing a conserved three-dimensional fold yet exhibiting diverse biological functions [13]. Unlike classical serpins, PEDF lacks protease inhibitory activity, implying that its effects are mediated primarily via interactions with cell-surface binding partners [14,15].

**Figure 1:**
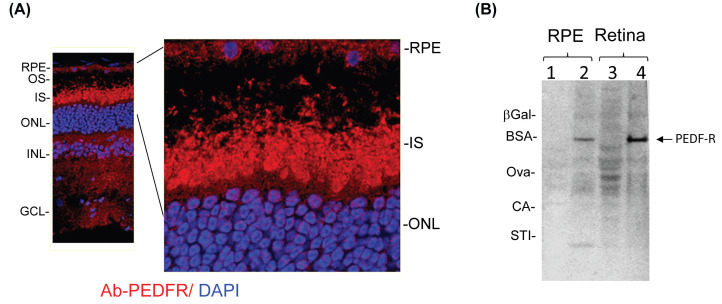
Distribution of PEDF-R in the bovine retina by immunoreactions with Ab-PEDF-R. (**A**) Immunofluorescence of PEDF-R (red) detected in the RPE and the inner segments (IS) of a section of bovine retina. RPE, retinal pigment epithelium; OS, outer segments of photoreceptors, IS, inner segments; ONL, outer nuclear layer; INL, inner nuclear layer, GCL, retinal ganglion cell layer. (**B**) Western blot of biochemical extracts of bovine RPE and retina. Lanes 1 and 3 correspond to cytosolic fractions, whereas lanes 2 and 4 correspond to membrane-enriched fractions. The arrow points to the protein corresponding to PEDF-R. PEDF-R is enriched in membrane-containing fractions from bovine retina and RPE extracts, consistent with membrane association in retinal cells.

Efforts to identify a PEDF-binding protein focused on membrane fractions from retinoblastoma cells, cerebellar granule neurons, and bovine retina using PEDF affinity chromatography [16,17]. A prominent protein band was consistently detected, but initially had no match in available databases. In the early 2000s, yeast two-hybrid screening in human fetal RPE cells identified this protein as the translational product of a previously uncharacterized transcript, later recognized as *PNPLA2* and the protein was detected in mammalian retina [4] (Figure 1).

In 2004, three independent groups cloned *PNPLA2* in non-retinal tissues, assigning distinct names reflecting the metabolic functions of its protein product: ATGL, desnutrin, and iPLA2ζ [5–7]. These studies established PNPLA2 as a key regulator of triglyceride hydrolysis in peripheral tissues. In 2006, PNPLA2 was reported to function as the PEDF-R, directly linking its phospholipase A2 enzymatic activity to PEDF-mediated cellular responses [4]. Subsequent investigations confirmed retinal expression and PEDF-dependent signaling in retinal cells [18], highlighting a direct mechanistic connection between lipid remodeling processes and photoreceptor survival pathways.

## Molecular and biochemical properties of PEDF-R

PEDF-R has been proposed to link extracellular neurotrophic signaling to intracellular lipid metabolism in retinal cells, functioning both as a receptor for PEDF and a multifunctional lipid-modifying enzyme. It exhibits phospholipase, triglyceride lipase, transacylase, and RE hydrolase activities [4–7]. Through PEDF binding, PEDF-R transduces survival and anti-apoptotic signals, while its enzymatic activities support lipid mobilization and membrane phospholipid remodeling [18,19]. These functions are regulated in part by PEDF itself, interacting proteins such as comparative gene identification 58 protein (CGI-58) [20] and G0/G1 switch gene 2 (G0S2) [21], and subcellular localization, allowing context-dependent control of retinal lipid metabolism and survival. While PEDF and CGI-58 are present in the RPE, G0S2 appears to be expressed at very low levels in the retina. Whether additional retinal-specific regulatory partners modulate PEDF-R activity remains unknown.

**Figure 2:**
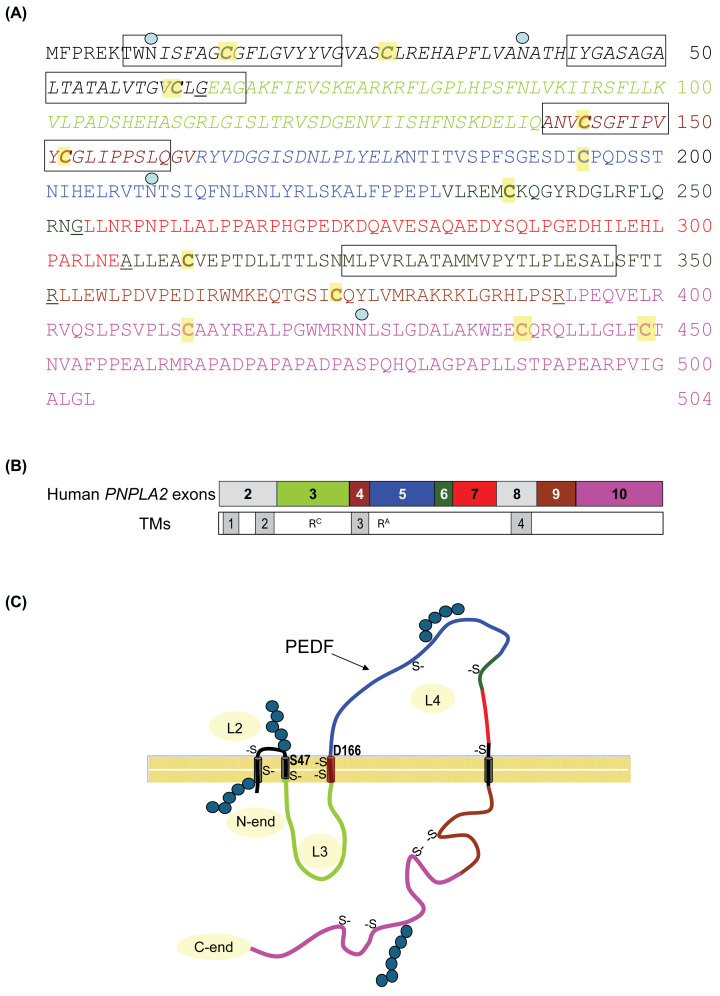
Exon organization and predicted structural features of human PEDF-R. (**A**) Amino acid sequence of human PEDF-R in single letter code. The PNPLA domain is in italics (Ile10-Lys179). Predicted transmembrane (TMs) domains are indicated by boxes. Predicted N-glycosylation consensus sites are shown by blue circles. The underlined amino acid is coded by a codon formed by junction. Cysteines are in bold and highlighted in yellow. The exon/intron structure reflects to a certain degree the proposed domain structure of the protein. TMs are flanked by introns. (**B**) Organization of translated exon sequences on the open reading frame (ORF) of human PEDF-R. The gene has 10 exons, and the ORF starts in exon 2. Exon sequences are color coded as in panel (A). In the second line, predicted TM domains are superimposed on the ORF exon organization. The ORF is indicated by an open box, the predicted TM domains by gray boxes that are aligned to the exon sequences. The location of peptides R^A^ (Cys194-Asn209), and R^C^ (Glu71-Arg79), epitopes of antibodies for intracellular and extracellular regions, respectively, are indicated. (**C**) Proposed membrane topology of PEDF-R based on computational prediction. Location of homologous PLA active sites S47 and D166 is indicated. Locations of cysteine are indicated by S-. Blue circles illustrate carbohydrates in N-glycosylation. Intracellular N- and C-end tails, intracellular and extracellular loops are indicated by yellow ovals. The net charge at pH 7.4 (z) these regions are: N-end, z = +0.275; Loop 2, z = −0.311; Loop 3, z = +2.768; Loop 4, z = −8.624; C-end, z = +1.882. The proposed extracellular interaction between PEDF and PEDF-R is illustrated schematically.

The human *PNPLA2* gene on chromosome 11p15.5 encodes a 504-amino-acid polypeptide (an expected molecular mass of 55,315.11 Daltons; GeneBank™, accession number BC017280). It contains ten exons and nine introns, with exon-intron junctions corresponding to functional domains, including the PNPLA catalytic domain (Ile10-Lys179) (Figure 2). Alternative splicing in retinal tissues produces isoforms with distinct enzymatic or localization properties [22]. Sequence analysis reveals four N-glycosylation sites and several cysteines that may contribute to folding. Recombinant PEDF-R produced in bacteria retains phospholipase activity despite the absence of glycosylation, indicating that glycosylation is not required for catalytic activity *per se*, although it may influence protein folding, stability, trafficking, or membrane localization in mammalian cells [4,5]. The catalytic dyad is formed by Ser47 (within the consensus GXSXG) and Asp166 (within the consensus DXG/A).

Phospholipase activity is particularly relevant in photoreceptors, which are rich in membrane phospholipids but low in triglycerides. PEDF binding stimulates basal phospholipase A_2_ activity, promoting the hydrolysis of phospholipids and the release of fatty acids such as docosahexaenoic acid (DHA). DHA can serve as a precursor for neuroprotectin D1 (NPD1), a bioactive lipid mediator promoting survival. PEDF stimulates NPD1 synthesis in RPE cells [23], and this response requires PEDF-R phospholipase activity [24]. Inhibition of PEDF-R activity by atglistatin, blocking PEDF binding, or PNPLA2 deficiency abolish PEDF-mediated cytoprotection [18,24]. Lipidomic analyses of PNPLA2-deficient retinas reveal DHA-containing lysophospholipid accumulation (LPC and LPE), consistent with its physiological substrates [25].

In the RPE, REs are stored in retinosomes—lipid bodies that serve as reservoirs for chromophore regeneration. When vitamin A is depleted, REs must be mobilized to produce 11-*cis*-retinal, the chromophore for visual pigments. PEDF-R/PNPLA2 is a major RE hydrolase contributing to mobilization of REs from retinosomes. It supports retinoid metabolism in the RPE, mobilizing REs for 11-*cis*-retinal production, sustaining the visual cycle [19]. The CGI-58 protein present in the RPE [26] interacts with and enhances the RE hydrolase activity of PEDF-R/PNPLA2 [19]. In the RPE, PEDF-R also contributes to phospholipid turnover during outer segment renewal by digesting phospholipids surrounding rhodopsin of outer segments of photoreceptors [27].

PEDF binding was mapped initially via yeast two-hybrid screening (Region 12, Gln250-Arg383) and later refined to extracellular segments (L4: Leu159-Met325; E5b: Ile193-Leu232, P1: Thr210-Leu249) [4,18]. These regions are distinct from the catalytic dyad, and truncation studies confirm the structural separability of ligand binding and enzymatic activity.

Collectively, PEDF-R is a multifunctional receptor-enzyme that integrates lipid remodeling with neurotrophic signaling. Its catalytic dyad, phospholipid-binding regions, and PEDF-interacting domains provide a framework for understanding function within specific retinal compartments. These molecular features provide a framework for understanding how the enzymatic and receptor functions of PEDF-R are arranged within specific retinal cell compartments, setting the stage for examining its cellular and subcellular localization in the retina.

## Retinal expression and subcellular localization of PEDF-R

The functional impact of PEDF-R in the retina depends not only on its biochemical properties but also on its spatial distribution within retinal cells. Photoreceptors and RPE cells are highly polarized and undergo continuous membrane turnover associated with photoreceptor outer-segment renewal and lipid recycling [28–30]. Therefore, the localization of PEDF-R within specific membrane domains influences both its catalytic activity and its accessibility to extracellular PEDF.

Computational analyses predict that PEDF-R contains multiple hydrophobic segments consistent with membrane insertion [4]. These regions likely form several membrane-spanning segments that generate extracellular loop domains and cytoplasmic N- and C-terminal regions (Figure 2). Mapping exon boundaries onto the amino acid sequence reveals that several predicted transmembrane segments are contained within individual exons (Figure 2), suggesting a modular structural organization of membrane-interacting regions. One extended extracellular loop spanning approximately Gln159–Asn324 contains the PEDF-binding sequences described above in the ‘Molecular and biochemical properties of PEDF-R’ section, consistent with the extracellular accessibility required for ligand recognition.

**Figure 3:**
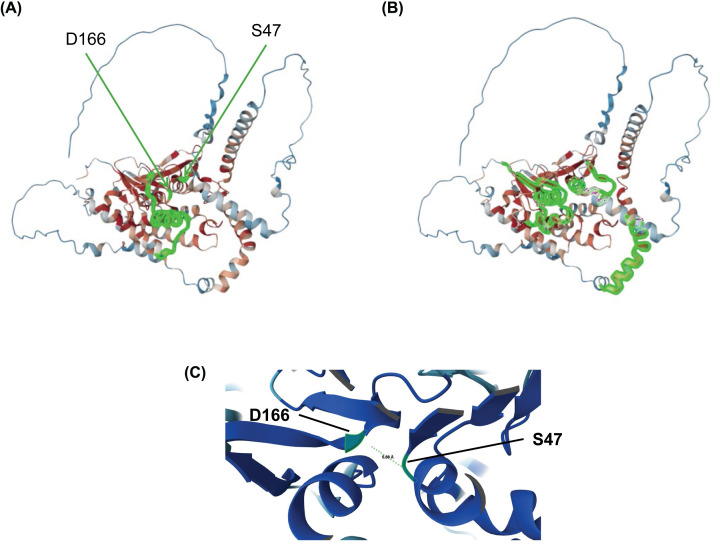
AlphaFold-predicted structure of human PEDF-R. (**A**,**B**) The AlphaFold-predicted structure is shown with indicated residues S47 and D166, corresponding to the putative catalytic dyad, and the proposed PEDF-binding region (Thr210–Leu232) highlighted in green (**A**). Predicted transmembrane segments are highlighted in green in panel (B). (**C**) Distance between S47 and D166 (5.88 Å) in the Alphafold-predictive model of PEDF-R.

Analysis of the ionic composition across predicted domains further supports this membrane orientation. The N-terminal cytoplasmic segment, a major intracellular loop, and the extended C-terminal region are enriched in basic residues relative to acidic residues. This distribution is consistent with the “positive-inside” rule described by von Heijne, whereby positively charged residues preferentially localize to the cytoplasmic side of biological membranes [31]. In contrast, the long extracellular loop contains a relatively higher proportion of acidic residues, further supporting the predicted topology. Structural models generated using AlphaFold indicate high confidence for the N-terminal catalytic domain but lower confidence for portions of the C-terminal region, suggesting conformational flexibility that may facilitate interactions with membranes or regulatory partners. In this predicted structure, the catalytic dyad and the PEDF-binding regions are distinct from the S47 and D166 (Figure 3). Because this model is based on AlphaFold predictions, structural features involving flexible loops and membrane-associated regions should be interpreted cautiously until validated experimentally. However, the structure of human PEDF-R protein remains to be resolved.

Experimental studies provide evidence for the presence of PEDF-R at the plasma membrane of retinal cells. Antibody accessibility assays in non-permeabilized cells demonstrate extracellular exposure of specific loop regions, consistent with surface localization [4]. Biochemical fractionation analyses similarly show that PEDF-R associates with membrane fractions rather than the soluble cytosol. These observations support the view that PEDF-R is a membrane-associated receptor capable of directly interacting with extracellular PEDF.

Although PEDF-R is accessible at the plasma membrane, its localization is not static. In triglyceride-rich tissues and cells, such as adipocytes, PEDF-R—also known as ATGL—localizes to the surface of lipid droplets, where it catalyzes triglyceride hydrolysis. Because lipid droplets are bounded by a phospholipid monolayer rather than a phospholipid bilayer [32], localization of PEDF-R to both lipid-droplet and plasma membranes suggests an ability to associate with diverse phospholipid-rich interfaces. In retinal cells, PEDF-R is primarily associated with plasma membranes but can redistribute to intracellular lipid compartments under lipid-enriched conditions in RPE cells [4,19,26]. This dual localization is consistent with the capacity of the enzyme to interact with both phospholipid bilayers and lipid-droplet monolayers.

These observations are consistent with a model in which PEDF-R may dynamically partition between membrane compartments. The protein may move between plasma membrane domains and intracellular lipid droplets or retinosome-like structures depending on metabolic conditions. The mechanisms controlling this redistribution remain incompletely understood but may involve changes in lipid composition, post-translational modifications, or interactions with regulatory partners.

Within the retina, this spatial distribution has important functional implications. Plasma membrane PEDF-R allows retinal cells to respond to extracellular PEDF, whereas association with intracellular lipid compartments supports lipid processing involved in photoreceptor outer-segment renewal and retinoid metabolism in the RPE. Through these activities, PEDF-R is thought to contribute to membrane phospholipid remodeling and to the mobilization of REs required for visual cycle function [19,27]. Therefore, the high membrane content of photoreceptor outer segments and lipid-rich phagosomes in the RPE position PEDF-R as a direct participant in processes that sustain retinal lipid homeostasis and visual transduction. These localization patterns provide the structural framework through which PEDF-R can influence photoreceptor survival, raising the question of how its receptor and enzymatic activities mechanistically contribute to photoreceptor homeostasis.

## Mechanistic roles of PEDF-R in photoreceptor homeostasis and aging

**Figure 4:**
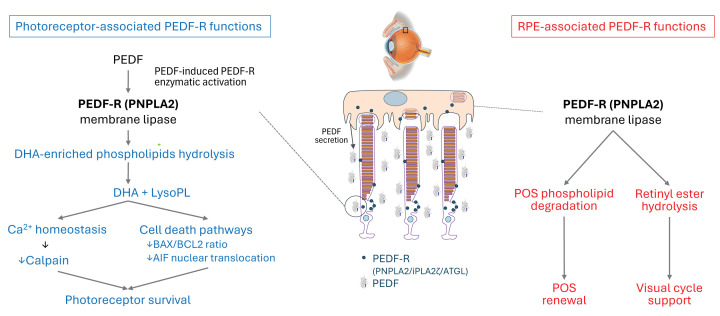
Summary of experimentally supported PEDF-R (PNPLA2) functions in photoreceptors and the RPE. The pathways shown summarize functional associations reported in multiple experimental systems and should not be interpreted as a single established signaling cascade or unified mechanistic pathway. PEDF secreted by the RPE binds to PEDF-R (PNPLA2) located at the photoreceptor plasma membrane. In photoreceptors (left, blue), PEDF binding is associated with activation of PEDF-R lipase activity, resulting in phospholipid hydrolysis and release of fatty acids and lysophospholipids. Because photoreceptor membranes are enriched in DHA-containing phospholipids, DHA is shown as a representative hydrolysis product, although PEDF-R can also hydrolyze phospholipids containing other fatty acids, including arachidonic acid. Experimental studies in photoreceptors have associated PEDF-R activity with modulation of calcium homeostasis, reduced calpain activation, altered expression of apoptosis-related proteins (BAX and BCL2), and decreased apoptosis-inducing factor (AIF) nuclear translocation. These responses are depicted as experimentally supported functional associations linked to photoreceptor survival. In the RPE (right, red), PEDF-R contributes to phospholipid degradation during phagocytic processing of photoreceptor outer segments and participates in RE hydrolysis within retinosomes, processes that contribute to outer-segment renewal and visual cycle function. Solid arrows represent experimentally supported functional associations.

Photoreceptor survival depends on the coordinated regulation of lipid turnover, metabolism, and stress-response pathways. PEDF-R has been implicated in these processes through its lipid hydrolase activity and its association with PEDF-dependent signaling. Experimental studies have associated PEDF-R with photoreceptor survival, membrane homeostasis, and stress responses, although the mechanistic relationships among these processes remain incompletely defined (Figure 4).

### Photoreceptor survival signaling

In photoreceptor models, PEDF binding to PEDF-R has been associated with activation of signaling pathways linked to cell survival, including STAT3 modulation, reduced AIF nuclear translocation, altered calcium homeostasis, and changes in apoptosis-related proteins such as BAX and BCL2 [33–36]. In retinal degeneration models, PEDF treatment reduced intracellular calcium and calpain activation, accompanied by decreased photoreceptor cell death [36]. These effects correlated with a reduced BAX/BCL2 ratio [36]. Importantly, inhibition of PEDF-R with atglistatin abolished the protective effects of PEDF and the H105A peptide, indicating that PEDF-R enzymatic activity contributes to these responses [24,34]. PEDF-derived peptides that retain PEDF-R binding activity preserve photoreceptor structure and function across multiple injury models [37–40]. Collectively, these findings are consistent with a role for PEDF-R in signaling pathways associated with photoreceptor survival, although the relationships among downstream events remain to be resolved.

### Lipid metabolism and membrane homeostasis

PEDF-R mediates phospholipid hydrolysis and lipid remodeling in retinal cells. In photoreceptors, outer segments undergo continuous renewal and require ongoing membrane turnover [29]. PEDF-R releases fatty acids, including DHA, and lysophospholipids from membrane phospholipids [24,25].

PEDF-R activity is not restricted to releasing DHA-containing phospholipids. Biochemical studies of PNPLA2/iPLA2ζ demonstrated hydrolysis of phospholipid substrates containing arachidonic acid as well as DHA [5]. Therefore, the biological consequences of PEDF-R activity may depend in part on the phospholipid composition of the membrane substrate available within a given cellular context. In photoreceptors, where DHA-containing phospholipids are highly enriched, PEDF-R activity would be expected to preferentially release DHA, potentially favoring generation of cytoprotective lipid mediators. Whether changes in retinal lipid composition during aging or disease alter the spectrum of lipid mediators released by PEDF-R remains to be determined.

DHA can serve as a precursor for NPD1, a cytoprotective lipid mediator. PEDF stimulates NPD1 synthesis in RPE cells, and studies in corneal epithelial cells indicate that this response requires PEDF-R phospholipase activity [23,24]. Whether a similar PEDF-R-dependent pathway operates directly in photoreceptors remains to be determined. Loss of PEDF-R activity has been associated with disrupted photoreceptor structure, reduced visual pigment content, and diminished electroretinographic responses in experimental models [19,25], consistent with a role for PEDF-R in maintaining membrane lipid homeostasis.

### Mitochondrial and stress-response associations

Signaling through PEDF/PEDF-R binding has been associated with mitochondrial-related stress responses in retinal models. PEDF treatment reduces AIF nuclear translocation and modulates BAX and BCL2 expression [35,36], suggesting effects on pathways involved in mitochondrial-associated cell death. Neurotrophic PEDF-derived peptides that bind PEDF-R have been shown to preserve mitochondrial activity in photoreceptors and promote cell survival and differentiation, whereas peptides derived from the antiangiogenic region of PEDF do not reproduce these effects [41]. Whether these observations reflect direct mitochondrial regulation or secondary effects of broader cellular stabilization remains unclear.

### RPE lipid processing functions

In the RPE, PEDF-R contributes to outer-segment degradation by supporting phospholipid hydrolysis during phagocytosis [27,42]. PEDF-R also participates in RE mobilization from retinosomes, supporting retinoid availability for the visual cycle [19]. These findings indicate roles for PEDF-R in RPE lipid and retinoid metabolism that indirectly support photoreceptor function.

### Aging and stress susceptibility

PEDF-R functions may become increasingly relevant during aging, when oxidative stress, inflammation, mitochondrial dysfunction, and altered lipid metabolism contribute to retinal vulnerability. PNPLA2 deficiency has been associated with AMD-like features and increased IL-6 expression in the RPE [43]. Reduced PEDF signaling has likewise been associated with impaired RPE function and senescence-related phenotypes, and PEDF deletion has been shown to reduce PEDF-R expression in the RPE [42,43], suggesting coordinated regulation of PEDF and PEDF-R that may influence photoreceptor outer segment phagocytosis and degradation.

Neurotrophic PEDF-derived peptides that bind PEDF-R confer protection against oxidative stress in human retinal organoid models, whereas non-binding peptides do not [37]. Additional studies have implicated PEDF-R signaling in pathways associated with glutathione metabolism, lipid peroxidation responses, and regulation of IL-6 production [40,43,44].

PEDF-R has also been reported to interact with 5-lipoxygenase (5-LOX), a key enzyme in leukotriene synthesis, and PEDF-R overexpression reduced leukotriene B4 production and attenuated oxidative stress-induced RPE cell death [45]. In the same study, oxidative stress reduced *PNPLA2* transcript levels without significantly affecting ALOX5 expression, suggesting that diminished PEDF-R expression may contribute to retinal stress susceptibility and inflammatory signaling through mechanisms extending beyond phospholipid hydrolysis.

Taken together, these findings suggest that PEDF-R contributes to pathways associated with retinal stress responses and cellular resilience. However, the relative contributions of lipid metabolic and signaling mechanisms remain incompletely understood.

Figure 4 summarizes experimentally supported PEDF-R functions and proposed mechanistic relationships in the photoreceptors and the RPE. The pathways are presented as associated processes derived from multiple experimental systems and should not be interpreted as a single established signaling cascade.

## Consequences of PEDF-R dysfunction in retinal degeneration

Loss of PEDF-R (PNPLA2) function is associated with structural and functional abnormalities in both photoreceptors and the RPE [19,25,27,43]. Experimental studies support roles for PEDF-R in retinal lipid metabolism and cellular homeostasis, although the relative contribution of individual mechanisms to disease progression remains incompletely defined.

### Photoreceptors

PNPLA2 deficiency has been associated with altered phospholipid composition, disrupted outer-segment organization, reduced visual pigment content, and mitochondrial abnormalities [25]. These changes are accompanied by impaired retinal function and progressive photoreceptor degeneration in experimental models. Collectively, these findings support a role for PEDF-R in maintaining the membrane lipid homeostasis required for photoreceptor structure and function, although whether mitochondrial and structural abnormalities arise through direct or indirect mechanisms remains unclear.

### RPE

In the RPE, PEDF-R deficiency impairs degradation of phagocytosed outer segments, leading to accumulation of lipid-rich material consistent with defective lipid processing [27]. PEDF-R deficiency also disrupts RE mobilization from retinosomes, resulting in accumulation of RE-containing lipid droplets and reduced retinoid availability for the visual cycle [19]. In addition, PNPLA2 deficiency has been associated with increased inflammatory signaling, including elevated IL-6 expression and AMD-like features in mice [43], suggesting broader effects on retinal homeostasis.

### Integrated interpretation

Collectively, these findings indicate that PEDF-R contributes to phospholipid turnover, retinoid metabolism, outer-segment processing, and stress responses in the retina. Disruption of these functions may increase susceptibility to retinal degeneration during aging and disease. However, the relationships among lipid dysregulation, oxidative stress, inflammation, and photoreceptor loss likely reflect the involvement of parallel and interacting pathways rather than a single linear mechanistic pattern.

## Conclusions and future perspectives

PEDF-R (PNPLA2) is a multifunctional receptor-enzyme that has been implicated in both lipid remodeling and trophic signaling pathways associated with photoreceptor structure and function. Its multifunctional enzymatic and receptor activities provide a useful framework for investigating mechanisms that contribute to photoreceptor homeostasis across the lifespan. Future studies should clarify the specific lipid substrates and downstream mediators that link enzymatic activity to survival-associated signaling, the mechanisms governing intracellular trafficking and compartmental plasticity in photoreceptors and RPE, and how age-dependent changes in PEDF/PEDF-R influence retinal resilience, including effects on expression, localization, and enzymatic activity. Another important area for future investigation is the identification of PEDF-R protein interaction networks in retinal cells. Beyond PEDF itself, PEDF-R activity is regulated by proteins such as CGI-58 and G0S2 in other tissues, and interactions with 5-lipoxygenase have been reported in RPE cells. Whether additional retinal-specific binding partners influence PEDF-R localization, substrate selection, signaling functions, or stress responses remains largely unexplored. Defining the retinal PEDF-R interactome may provide new insight into how lipid metabolism, inflammation, and cell-survival pathways are coordinated in photoreceptors and the RPE. Additionally, exploration of PEDF-derived peptides or small molecules that enhance PEDF-R activity may offer opportunities to preserve photoreceptor function in aging or disease.

PEDF-based therapeutic strategies, ranging from gene therapy approaches currently under clinical evaluation for retinitis pigmentosa [3] to PEDF-derived H105A eye drops that have demonstrated robust preclinical efficacy and have been identified as candidates for future clinical translation [37], underscore the clinical promise of PEDF-mediated neuroprotection and the potential of PEDF-PEDF-R signaling as a therapeutic target. Addressing these mechanistic questions will be essential for advancing PEDF-R-targeted interventions for retinal degenerative diseases.

## Open Access

Open access for this article was enabled by the participation of the National Institutes of Health (NIH) in an all-inclusive Read & Publish agreement with Portland Press and the Biochemical Society.

## Abbreviations

AIF: apoptosis-inducing factor
BCL2: B-cell lymphoma 2
BAX: BCL@-associated X protein
DHA: docosahexaenoic acid
NPD1: neuroprotectin D1
ORF: open reading frame
PEDF: pigment epithelium-derived factor
PEDF-R: PEDF receptor
PNPLA: patatin-like phospholipase A
RE: retinyl ester
RPE: retinal pigment epithelium
serpin: serine protease inhibitor
TM: transmembrane
